# Behavior of Complement System Effectors in Chronic and Acute Coronary Artery Disease

**DOI:** 10.3390/jcm14113947

**Published:** 2025-06-03

**Authors:** Roxana Mihaela Chiorescu, Mihaela Mocan, Maria Iacobescu, Cristina Adela Iuga, Dan Blendea, Horia Stefan Roșian, Raluca Mihaela Tat, Edina Mate, Horea Rus, Sonia Irina Vlaicu

**Affiliations:** 1 Internal Medicine Department, “Iuliu Hatieganu” University of Medicine and Pharmacy, 400012 Cluj-Napoca, Romania; roxana.chiorescu11@gmail.com (R.M.C.); vlaicus@yahoo.com (S.I.V.); 2 Department of Internal Medicine, Emergency Clinical County Hospital, 400006 Cluj-Napoca, Romania; 3 Department of Proteomics and Metabolomics, Research Center for Advanced Medicine—MEDFUTURE, “Iuliu Hatieganu” University of Medicine and Pharmacy Cluj-Napoca, 400349 Cluj-Napoca, Romania; ilies.maria@umfcluj.ro (M.I.); iugac@umfcluj.ro (C.A.I.); 4 Department of Pharmaceutical Analysis, Faculty of Pharmacy, “Iuliu Hatieganu” University of Medicine and Pharmacy, 400349 Cluj-Napoca, Romania; 5 Department of Cardiology, “Nicolae Stăncioiu” Heart Institute, 400001 Cluj-Napoca, Romania; dblendea1@gmail.com (D.B.); dr.rosianu@gmail.com (H.S.R.); 6 Department of Cardiology, “Iuliu Hatieganu” University of Medicine and Pharmacy, 400437 Cluj-Napoca, Romania; 7 Department of Surgery, “Iuliu Hatieganu” University of Medicine and Pharmacy, 400012 Cluj-Napoca, Romania; raluca.tat@umfcluj.ro; 8 Department of Emergency Medicine, Emergency Clinical County Hospital, 400006 Cluj-Napoca, Romania; 9 Department of Neurology, University of Maryland School of Medicine, Baltimore, MD 21201, USA; hrus@umaryland.edu; 10 Neurology Department, Baltimore Veterans Administration Hospital, Baltimore, MD 21201, USA

**Keywords:** RGC-32, SIRT1, complement SC5b-9, atherosclerosis, vulnerable atherosclerotic plaque

## Abstract

**Background/Objectives:** The complement system (particularly C5b-9) is an instrumental part of the induction and progression of atherosclerosis. The fluid phase C5b-9, also known as soluble C5b-9 (sC5b-9), is a reliable indicator of terminal complement pathway activation. Response Gene to Complement (RGC)-32 is a C5b-9 effector involved in cell cycle regulation and differentiation, immunity, tumorigenesis, obesity, and vascular lesion formation. RGC-32 regulates the expression of Sirtuin1 (SIRT1), known to delay vascular aging. The aim of this study was to assess the levels of sC5b-9, RGC-32, and SIRT1 in patients with atherosclerotic chronic and acute ischemic coronary syndromes. **Methods:** We determined the levels of sC5b-9, serum RGC-32, and SIRT1 by enzyme-linked immunosorbent assays (ELISAs) in 41 patients with chronic atherosclerotic coronary syndromes, 36 patients with acute ischemic coronary syndromes, and 21 asymptomatic controls with no history of ischemic heart disease. **Results:** sC5b-9 was significantly higher in patients with acute coronary syndrome as compared to the control group (*p* = 0.020, AUC = 0.702). In chronic coronary ischemia patients, serum RGC-32 was correlated with the extension of coronagraphically visualized atherosclerotic lesions (r = 0.352, *p* = 0.035) as well as with sC5b-9 levels (r = 0.350, *p* = 0.025). RGC-32 concentration was significantly lower in patients with acute coronary syndrome than in the control group (*p* = 0.020). We also observed significantly lower serum SIRT1 concentrations in patients with chronic ischemic heart disease than in the control group (*p* = 0.025). **Conclusions:** sC5b-9 may function as a possible biomarker for myocardial tissue damage in acute coronary syndrome. In acute coronary syndrome settings, low levels of RGC-32 may indicate a protective, antifibrotic function of RGC-32 in the ischemia-damaged myocardium; however, in stable chronic disease, RGC-32 serum values appear to correlate with the extent of atherosclerotic lesions, suggesting a pro-atherogenic role for RGC-32. Chronic myocardial ischemia decreases SIRT1 protein levels in serum, which underscores the use of SIRT1-modulating drugs in these patients.

## 1. Introduction

Cardiovascular disease (CVD) is the leading cause of morbidity and mortality worldwide, affecting over 523 million people globally. CVD burden is heavily influenced by atherosclerotic diseases, with half of CVD deaths being attributed to ischemic heart disease and another quarter to ischemic stroke [1]. The absence of *in vivo* models that develop the disruption of atherosclerotic plaques has impeded progress in the mechanistic research on atherothrombosis [2]. Diagnosing a vulnerable plaque phenotype well before fatal clinical endpoints are reached is a challenge for personalized medicine [3,4]. In the case of anti-atherosclerotic therapies, even when an excellent control of dyslipidaemia is obtained, other sources of risk persist, in the form of the continuous activation of immune-inflammatory pathways [5,6]. There is a great need to identify new targets for anti-inflammatory therapeutic interventions, leading to a reduction in the residual inflammatory risk [7,8]. Identifying and characterizing several biomarkers [9] with prognostic value in CVD, such as serum values of apelin-13 [10] or sST2 [11], has been the subject of numerous studies in recent years.

As part of innate immunity, the complement system (especially C5b-9) is involved in both the induction and the progression of atherosclerosis [12,13]. Experimental studies have outlined a substantial role for the complement system in atherosclerosis: the proximal classical complement pathway apparently contributes to a protective effect, whereas distal complement activity accelerates atherogenesis [14]. Recent data point to the liver-independent, cell-autonomous, and non-canonical complement activities as being unacknowledged promoters of atherosclerosis [15]. The assembly of sublytic C5b-9 in the atherosclerotic plaque results in the activation and proliferation of smooth muscle and endothelial cells [14].

The Response Gene to Complement (RGC)-32 gene was first cloned from rat oligodendrocytes by differential display as part of an endeavor to identify the genes differentially expressed in response to complement activation [16,17]. In human, aortic smooth muscle cells (SMCs), sublytic C5b-9 induces the expression of RGC-32, and RGC-32 overexpression induces cell cycle progression [16]. In addition to its role in cell cycle activation and differentiation, RGC-32 is also involved in immunity, tumorigenesis, obesity, diabetes mellitus, atherosclerosis, and multiple sclerosis (MS) [17,18]. The experimental evidence argues for a pro-atherogenic role for RGC-32: ApoE^−/−^ RGC32^−/−^ mice display a diminished formation of atherosclerotic lesions when compared to ApoE^−/−^ control mice [19]. RGC-32 contributes to the formation of atherosclerotic vascular lesions by mediating the proliferation, migration [20], and differentiation [17,21] of aortic SMCs.

RGC-32 has been shown to be present in MS brain tissue/MS plaques; statistically significantly lower levels of RGC-32 mRNA expression have been documented in the peripheral blood monocytes (PBMCs) of MS patients with relapses as compared to those who are clinically stable [17]. RGC-32 mRNA levels are significantly higher in patients defined as responders compared to non-responders to glatiramer acetate (GA), and RGC-32 levels decline in patients experiencing clinical relapses, all of which warrants future use of RGC-32 as a serum biomarker for the detection of MS patient relapse and response to GA therapy [17].

Plasma complement component C5 was shown to function as a novel biomarker of subclinical atherosclerosis [22], while sC5b-9 was identified as a potential biomarker for predicting the severity and stability of carotid plaques in acute ischemic stroke patients [23].

RGC-32 regulates the expression of Sirtuin1 (SIRT1) in several tissues [17]. SIRT1, a member of the sirtuin family, increases nitric oxide (NO) production, reduces inflammation and oxidative stress, induces autophagy, and prevents senescence, all of which contribute to slowing the progression of atherosclerosis and vascular aging [24]. SIRT1 expression in PBMCs isolated from patients with stable coronary artery disease (CAD) and acute coronary syndromes is reduced as compared to subjects without angiographically demonstrable CAD [25].

The objective of this study was to evaluate the serum concentrations of sC5b-9, RGC-32, and SIRT1 histone deacetylase in patients with atherosclerotic ischemic coronary disease and to verify their reliability as serum biomarkers for acute or for chronic coronary syndromes. Our working hypothesis was that levels of sC5b-9, RGC-32, and SIRT1 differ according to with plaque instability among acute coronary syndromes, chronic coronary syndromes, and control patients.

## 2. Materials and Methods

Study Population:

We conducted a prospective, observational, analytical case–control group study lasting one year. Ninety-eight patients hospitalized in the Cardiology Ward of an Emergency Clinical County hospital (ECCH) during a 12-month period (1 June 2023–1 July 2024) were included in this study, with the approval of the Bioethics Commission of “Iuliu Hațieganu” University of Medicine and Pharmacy in Cluj-Napoca (No. 237/30.08.2022). All patients included in this study provided their written consent for participation [26].

Research Location:

Patient recruitment, clinical assessment, biological sample collection, and database recording/completion took place at the Medical Clinic 1—ECCH Cluj-Napoca, Internal Medicine 1 Department, and at the “Nicolae Stăncioiu” Heart Institute Cluj-Napoca. Serum processing for general parameters (total cholesterol, triglycerides, HDL cholesterol, LDL cholesterol, uric acid, urea, creatinine, sodium, potassium, glucose, glycated hemoglobin, hemoglobin, hematocrit, platelets, hs-CRP, troponin I [TnI], creatine kinase, creatine kinase-MB, and transaminases) was conducted at the Central Laboratory of SCJU Cluj-Napoca and the Laboratory of the “Nicolae Stăncioiu” Heart Institute, Cluj-Napoca. Sandwich ELISA determinations were carried out at the Advanced Medicine Research Center.

Inclusion Criteria: Patients were divided into three groups:

(1) Patients with acute coronary syndrome (ACS) without ST-segment elevation (NSTEMI Group): 36 patients with unstable angina (UA) or non-ST-segment elevation myocardial infarction (NSTEMI) hospitalized in the Internal Medicine I and Cardiology I Departments of ECCH Cluj. The inclusion criteria for this group included meeting the criteria for UA/NSTEMI according to the current ESC 2023 guidelines [27], age over 18 years, and agreement to participate in this study. Symptoms of myocardial ischemia included: angina-like chest pain at rest, lasting more than 20 min; de novo angina-like pain; and destabilization of previously stable effort angina (crescendo angina). EKG changes during an acute episode included: ST-segment depression of greater than 0.5 mm, horizontal or descending; transient ST-segment elevations; T-wave inversions or flattening; and pseudo-normalization of previously inverted T waves.

(2) Patients with angina pectoris stable (APS) (chronic coronary syndromes) (APS group): This group consisted of 41 patients with stable angina hospitalized in the Internal Medicine I and Cardiology I Departments of ECCH Cluj. The inclusion criteria for this group included meeting the criteria for stable angina according to the ESC 2019 guidelines [28], age over 18 years, and agreement to participate in this study. Patients with symptoms of typical or atypical angina CCS I-III were included in this study. Resting EKG changes for the patient were: ST-segment depression of greater than 0.5 mm, horizontal or descending, transient ST-segment elevations, T-wave inversions or flattening, and pseudo-normalization of previously inverted T waves or normal EKG. In addition, the patients should not have changes in troponin level I.

(3) Control group (CO): This group consisted of 21 asymptomatic patients with no history of acute or chronic coronary syndrome evaluated in outpatient or during hospitalization in the Internal Medicine I or Cardiology I Departments of ECCH Cluj.

Exclusion Criteria: Presence of ST-segment elevation on EKG, moderate or severe valvular heart disease, prior surgical interventions for the correction of valvular disorders, infective endocarditis, spontaneous coronary dissection, Takotsubo syndrome, autoimmune diseases, bacterial infection in the last 6 weeks, hereditary or acquired angioedema, acute or chronic kidney disease, and acute inflammatory disease or neoplastic disease.

Study Protocol:

All patients were evaluated twice: once during their first admission and again after a period of 6 months. In both evaluations, the same parameters were recorded, including clinical, biological, electrocardiographic, and echocardiographic data.

For biological investigations, blood was collected via venous puncture under sterile conditions and with minimal venous compression. Biological assays were performed using the KONELAB 30 I analyzer (ThermoScientific, Waltham, MA, USA), measuring the following parameters: blood glucose, HDL cholesterol, LDL cholesterol, uric acid, triglycerides, C-reactive protein, creatinine clearance (mL/min), and corrected creatinine clearance (mL/min/1.73 m^2^). Troponin samples were collected upon the patients’ presentation with ACS and were repeated after 2 h and 8–12 h on the first day of hospitalization. The serum was collected within the first 12 h after symptom onset in NSTEMI patients and at the time of presentation in the APS patients and control groups. The serum was then stored at −80 °C and later used to measure RGC-32, SIRT1, and sC5b-9 levels by sandwich enzyme-linked immunosorbent assays (ELISAs).

Each sample was analyzed in duplicate according to the instructions provided with the respective kits. RGC-32 was measured using kits from ELK Biotechnology (Denver, CO, USA, catalog number ELK6515) and Elabscience Biotechnology (Houston, TX, USA, catalog number E-EL-H5305). SIRT1 was measured using an Elabscience Biotechnology kit (Houston, TX, USA, catalog number E-EL-H1546); the sC5b-9 kit was from Elabscience Biotechnology (Houston, TX, USA, catalog number E-EL-H2376).

A calibration curve was generated for each parameter using the protein standards provided in the kits. Absorbance readings were obtained with a ClarioStar microplate reader (BMGLabtech, Ortenberg, Germany), and data acquisition and processing were performed using integrated Mars 3.1 software. Quantification was based on a four-parameter fit calibration curve, and final concentrations were calculated as the mean of the two measurements.

Electrocardiograms were performed upon admission in patients with ACS, repeated dynamically in emergencies, and subsequently at 6 h intervals within the first 24 h, continuing daily during hospitalization and during angina crises. Patients were re-evaluated clinically and with EKG at 6 months. For patients with APS, EKGs were performed at presentations in the outpatient clinic and followed up at 6 months. Repeated EKGs were conducted in the event of recurrent angina during this 6-month interval.

An echocardiography was performed for each patient using a 2–5 MHz transducer on a Siemens Acuson X300 (Siemens Healthineers, Munich, Germany) ultrasound machine, evaluating left ventricular (LV) structural and performance parameters. The echocardiographic examination focused primarily on assessing wall motion and any potential diastolic and systolic dysfunction. Measurements included the end-systolic and end-diastolic diameters of the left ventricle (EDD, ESD). Normal values from the literature for healthy adults were considered to be: 38–56 mm for the EDD and 18–40 mm for the ESD.

Coronary angiography was performed within the first 24 h in patients with ACS and in the last 6 months before presentation in patients with APS. The vascular ultrasound examination conducted upon patient enrolment into this study focused on determining the intima-media thickness (IMT) and assessing the presence of atherosclerotic plaques with significance of extra coronary atherosclerotic expansion. The severity of coronary stenosis was assessed by coronary angiography, based on the visual estimation of the degree of arterial lumen narrowing, independently performed by two experienced interventional cardiologists. In case of discrepancy, consensus was reached through the joint review of the angiographic images.

Significant coronary stenosis was defined as a ≥50% narrowing of the luminal diameter of major epicardial vessels, according to a visual angiographic evaluation. Patients were categorized into three groups, single-vessel disease (1VD), two-vessel disease (2VD), and three-vessel disease (3VD), based on the number of major vessels (LAD, Cx, RCA) affected by significant lesions. Left, main, coronary artery lesions were considered equivalent to two- or three-vessel disease, depending on the extent of distal branch involvement [28].

The evolution was considered unfavorable when patients presented with complications during hospitalization (arrhythmias, left ventricular failure, and inadequate response to depleting therapy), readmission owing to cardiac decompensation, or death.

Statistical analysis was conducted using the IBM SPSS 26 software package. The data were analyzed descriptively and prescriptively. Since the data were not normally distributed, non-parametric tests were used.

Continuous variables were summarized as mean ± standard deviation (SD) and compared across groups using one-way analysis of variance (ANOVA), except for the number of affected coronary arteries, which was compared between the APS and NSTEMI groups using the non-parametric Mann–Whitney U test. Categorical variables were presented as frequencies and percentages and compared using the chi-squared test of independence. Group comparisons were conducted across three predefined groups: Control, APS, and NSTEMI. All tests were two-tailed, and a *p*-value < 0.05 was considered statistically significant. Statistical analyses were performed using Python 3.12 version (pandas, scipy).

Prior to the main analysis, the dataset underwent a cleaning process to ensure the robustness of the statistical tests. Outliers were identified and removed from three key variables: SIRT1, RGC-32, and sC5b-9. This was performed using the quartile rule by calculating the interquartile range (IQR) for each variable and then excluding any data point that fell more than 1.5 times the IQR above the third quartile or below the first quartile.

Data cleaning ensured that the subsequent statistical tests were less likely to be influenced by extreme values, providing a more accurate representation of the central tendencies within each experimental group.

After cleaning, the assumption of normality was tested for each group (control, APS, and NSTEMI groups) using the Kolmogorov–Smirnov and Shapiro–Wilk tests.

The Kolmogorov–Smirnov results for the control group showed non-normality for RGC-32, D(22) = 0.365, *p* < 0.001, and SC5b-9, D(22) = 0.272, *p* < 0.001. The Shapiro–Wilk test results were consistent with these findings: RGC-32: W(22) = 0.641, *p* < 0.001; sC5b-9: W(22) = 0.822, *p* = 0.001. SIRT1 was also found to be non-normally distributed: W(21) = 0.899, *p* = 0.034.

For the APS group, the Kolmogorov–Smirnov test indicated that RGC-32 scores were not normally distributed: D(40) = 0.185, *p* = 0.001, as well as sC5b-9, D(38) = 0.143, *p* = 0.048. The Shapiro–Wilk test also confirmed non-normality for these variables: RGC-32 W(40) = 0.831, *p* < 0.001; sC5b-9: W(38) = 0.907, *p* = 0.004. SIRT1 approached non-normality: W(36) = 0.939, *p* = 0.048.

For the NSTEMI group, both tests indicated non-normal distributions for all variables: SIRT1, D(34) = 0.257, *p* < 0.001, W(34) = 0.774, *p* < 0.001; RGC-32, D(36) = 0.187, *p* = 0.003, W(36) = 0.818, *p* < 0.001; SC5b-9, D(35) = 0.193, *p* = 0.002, W(35) = 0.851, *p* < 0.001.

All *p*-values reported are two-tailed. The tests utilized Lilliefors significance correction and showed that the data did not follow a normal distribution in any of the groups for the variables tested. Consequently, non-parametric tests were deemed appropriate for subsequent analyses.

After cleaning, the data were found to be adequately prepared for the Kruskal–Wallis H tests, which assessed differences between the experimental groups with the revised datasets. The significance level was set at 0.05 for all tests. Adjusted significance levels were applied to post hoc comparisons using the Bonferroni correction method.

The receiver operating characteristic curve (ROC) was used to assess the accuracy of the tests using the prediction variables considered. The predictive capacity of each variable was determined by the analysis of the area under the curve (AUC).

The Youden Index (YI) was used to determine the cut-off value [29]. The index was determined using the formula YI = Sensitivity + Specificity − 1.

## 3. Results

### 3.1. The Main Clinical, Electrocardiographic, Cardiac Ultrasound, Coronary Angiography, Blood Biochemical Parameters and Therapeutic Data for the Three Groups of Patients (NSTEMI, APS, and Control Groups)

The related data are presented in Table 1.

We set out to compare the levels of sC5b-9, RGC-32, and SIRT1 in the three groups of patients in order to identify biomarkers of unstable plaque and their role in atherosclerosis. ROC was used to assess the accuracy of the tests, using the prediction variables considered.

Our post hoc analysis indicated that sC5b-9, with the Kruskal–Wallis Statistic = 7.505, had a power of approximately 52.7%; RGC-32 with the Kruskal–Wallis Statistic = 7.400 had a power of approximately 0.520; and SIRT1 with the Kruskal–Wallis Statistic = 8.999 had a power of approximately 0.621. This indicates a relatively moderate probability of detecting the true effects. However, this post hoc analysis has limited value for justifying the a priori sample size.

### 3.2. sC5b-9 as a Biomarker of Coronary Plaque Vulnerability

**Figure 1:**
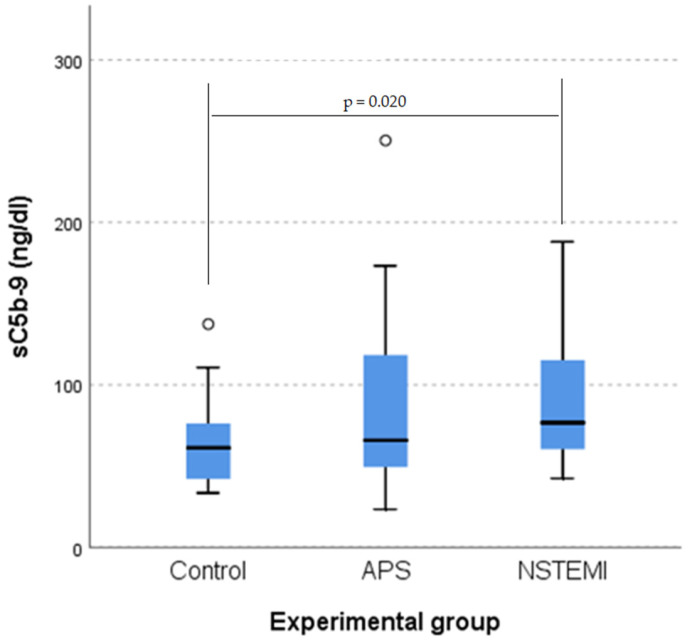
Boxplot of sC5b-9 value distributions for the three categories of patients (**left**). Outliers are marked with a circle. The statistically significant differences between groups were confirmed by the Kruskal–Wallis test (**right**).

A Kruskal–Wallis H test indicated a statistically significant difference in sC5b-9 levels across groups. (χ^2^(2) = 7.505. *p* = 0.023). Post hoc analysis showed a significant difference between the control group and the non-ST-elevation myocardial infarction (NSTEMI) group (*p* = 0.020) but not between the APS and NSTEMI groups (*p* = 0.411), or between the control and APS groups (*p* = 0.369) after applying Bonferroni’s correction. sC5b-9 scores were significantly higher for the NSTEMI group (mean rank = 57.00) than for the control group (mean rank = 35.75 (Figure 1)).

**Figure 2:**
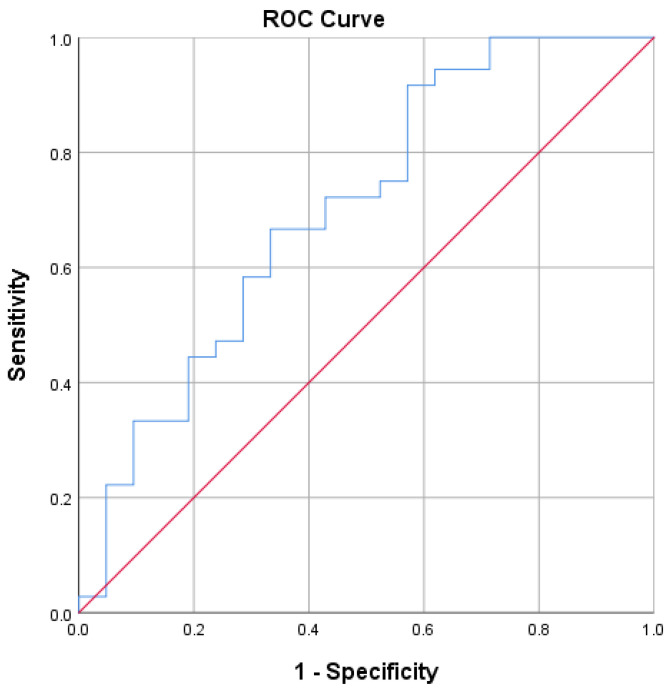
Predictive ability of sC5b-9 to differentiate patients with NSTEMIs from the control group. ROC curves: The blue curve depicts the performance of sC5b-9 as a biomarker, while the red diagonal line represents random classification. The area under the curve (AUC) is 0.702, indicating a moderate discriminative ability of sC5b-9 in distinguishing NSTEMI patients from controls.

An ROC curve analysis was conducted to evaluate the diagnostic ability of sC5b-9 levels to distinguish between patients with a non-ST-elevation myocardial infarction (NSTEMI) and the control group (Figure 2).

The area under the curve (AUC) for sC5b-9 was 0.702, suggesting a moderate ability to distinguish between NSTEMI patients and the control group. The 95% confidence interval for the AUC ranged from 0.557 to 0.848, indicating statistical significance, with the AUC being significantly different from 0.5 (*p* = 0.011). The Youden Index (YI) was used to determine the cut-off value. In this case, a YI of 0.346 indicated an optimal cut-off value for the sC5b-9 test result variable based on maximizing a YI of 54.40 ng/mL. At this value, the sensitivity is 0.917 and the specificity is 0.429.

Therefore, sC5b-9 levels were able to differentiate patients with acute coronary syndromes from those in the control group. As a marker of inflammation, an increase in sC5b-9 indicated that the vulnerability of the atherosclerotic plaque responsible for acute coronary syndromes is strongly linked to inflammation, making sC5b-9 a potential marker for plaque vulnerability.

### 3.3. RGC-32 Is Significantly Lower in Patients with NSTEMI

**Figure 3:**
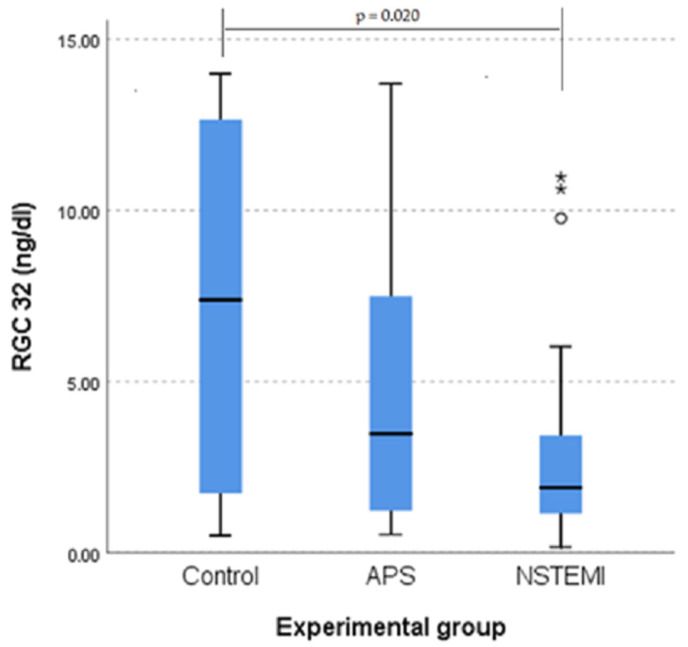
RGC-32 value distributions for the three categories of patients (**left**). Outliers in the boxplot are marked with a circle, and extreme outliers with an asterisk. The statistically significant difference between groups was confirmed by a Kruskal–Wallis test (**right**).

A Kruskal–Wallis H test showed a statistically significant difference in RGC-32 scores between the groups (χ^2^(2) = 7.400, *p* = 0.025). Post hoc tests revealed significant differences between the NSTEMI group and the control group (*p* = 0.020), but no significant difference between the APS and NSTEMI groups (*p* = 0.454) or between the APS and control groups (*p* = 0.337) after applying Bonferroni’s correction. RGC-32 scores for the NSTEMI group were significantly lower (mean rank, 38.26) than those of the control group (mean rank, 58.98) (Figure 3).

We also examined the possible correlation of RGC-32 values with inflammation biomarkers (sC5b-9) and the extension of atherosclerosis.

#### 3.3.1. Correlation Between RGC-32 and sC5b-9 in Patients with APS

**Figure 4:**
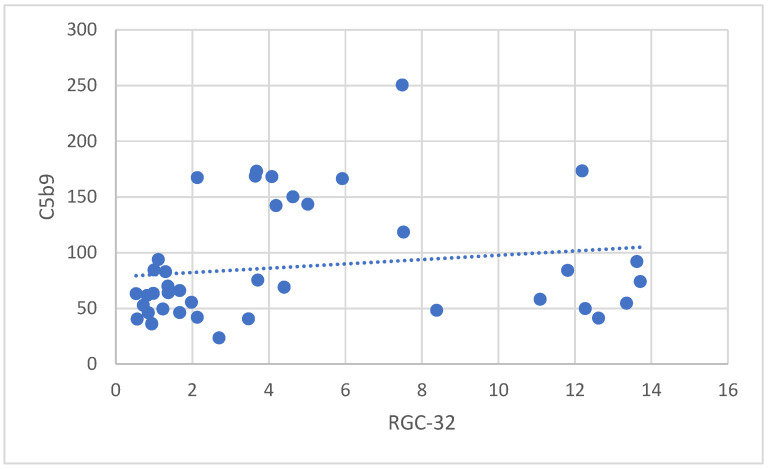
Correlation between RGC-32 and sC5b-9 in patients with APS.

Spearman’s rank-order correlation was conducted to determine the relationship between RGC-32 and sC5b-9 values in the APS group. The analysis revealed a moderate, positive correlation between RGC-32 and sC5b-9, which was statistically significant (r_s(41) = 0.350), (*p* = 0.025). This result suggests that higher RGC-32 values are associated with higher sC5b-9 values (Figure 4).

No other significant associations were found between proteins in the other groups.

#### 3.3.2. Correlation Between RGC-32 and the Extension of Atherosclerosis in Patients with APS

Our analysis revealed a moderate, positive correlation between RGC-32 values and the number of affected coronary arteries in the APS group, a finding that was statistically significant (r_s (36) = 0.352, *p* = 0.035). This means that higher RGC-32 values were associated with a greater number of affected coronary arteries, a key finding of our study.

No other significant associations were found between proteins in the three groups.

The significant correlations found suggest that, in the APS group, higher values of RGC-32 are associated with higher values of sC5b-9 and a higher number of affected coronary arteries. These relationships highlight a potential link between RGC-32 levels and these clinical parameters within this specific group of patients and underscore the importance of our findings in understanding and potentially treating APS.

#### 3.3.3. RGC-32 in the Presence vs. Absence of Myocardial Infarction in Patients with APS

A Mann–Whitney U test was conducted to determine whether there were differences in RGC-32 values between respondents who had experienced a myocardial infarction (Yes) and those who had not (No) within the APS group. RGC-32 values were statistically significantly lower in respondents who had experienced a myocardial infarction (mean rank = 17.38) than in those who had not (mean rank = 25.70), (U = 109.500), (z = −2.179), (*p* = 0.029), (*p* = 0.028).

**Figure 5:**
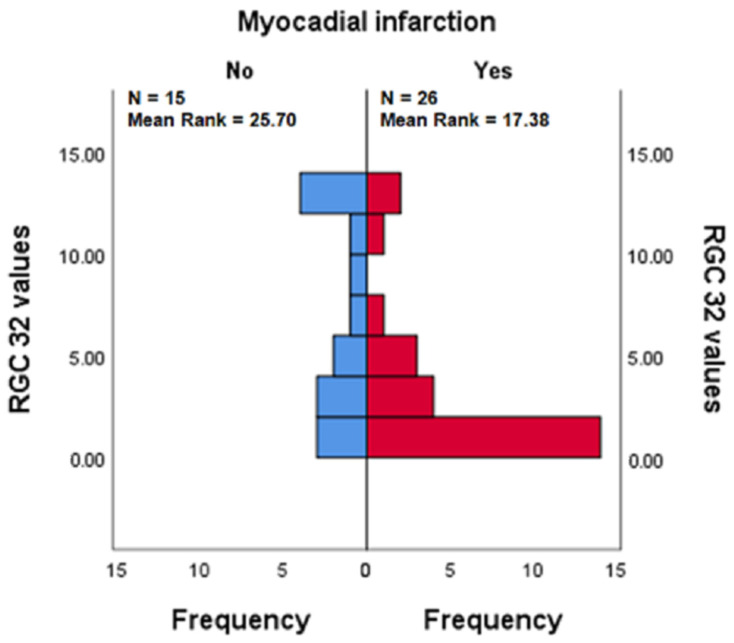
RGC-32 levels in patients with and without myocardial infarction among those with APS. The statistically significant difference was confirmed by the Mann–Whitney test.

The results suggest that experiencing a myocardial infarction is associated with lower RGC-32 values in the APS group. It is worth noting that no other significant differences were observed between respondents who had experienced a myocardial infarction in the NSTEMI group for any of the tested proteins (Figure 5).

The decrease in RGC-32 values in patients with myocardial infarction was consistent with the previous studies showing that RGC-32 plays an antifibrotic role and that RGC-32 decreases allow collagen accumulation [30].

No correlation was found between the values of the studied parameters and other characteristics of the patients: age, sex, presence/absence of hypertension, presence/absence of diabetes, total cholesterol value, LDL cholesterol value, HDL cholesterol value, triglyceride value, LV ejection fraction, or presence of heart failure.

To further explore these associations, a binary logistic regression analysis was conducted to assess the impact of the aforementioned clinical and demographic factors on the likelihood of myocardial infarction. The overall model was not statistically significant, χ2(8) = 12.281, *p* = 0.139, indicating that the included variables did not significantly predict the occurrence of myocardial infarction. The Nagelkerke R^2^ value of 0.193 suggests that only 19.3% of the variance in myocardial infarction status was explained by the model.

None of the individual predictors reached statistical significance at the 0.05 level. However, the presence of heart failure was the closest to significance, with a *p*-value of *p* = 0.092 and an odds ratio of 0.320 (95% CI: 0.085–1.204). Other variables, including age (*p* = 0.518), sex (*p* = 0.344), hypertension (*p* = 0.225), diabetes (*p* = 0.901), LDL cholesterol (*p* = 0.491), HDL cholesterol (*p* = 0.244), and triglycerides (*p* = 0.354), did not show significant associations with myocardial infarction status.

### 3.4. SIRT1 Plays a Role in Protection Against Atherosclerosis

**Figure 6:**
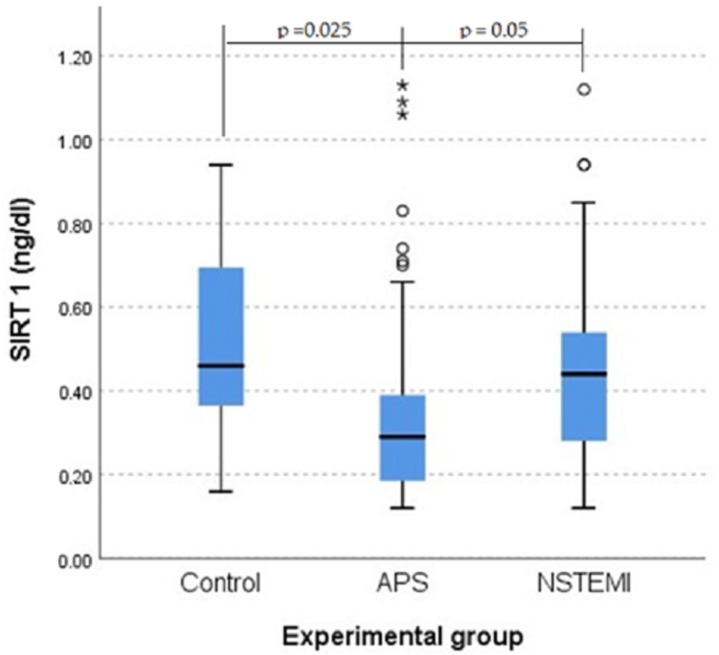
Boxplot of SIRT1 value distributions for the three categories of patients. The statistically significant difference between groups was confirmed by a Kruskal–Wallis test. Outliers are marked with a circle, and extreme outliers with an asterisk.

A Kruskal–Wallis H test showed a statistically significant difference in SIRT1 scores between the groups (χ^2^(2) = 8.999, *p* = 0.011). Post hoc tests revealed significant differences between the APS group and the control group (*p* = 0.025) and a difference between the APS group and the NSTEMI group (*p* = 0.05), but no significant difference between the NSTEMI and control groups (*p* = 1.000) after applying Bonferroni’s correction. APS mean scores (mean rank = 36.63) were significantly lower than the scores for the control group (mean rank = 56.08) (Figure 6).

The pathophysiology of acute coronary syndromes differs from the pathophysiology of chronic coronary syndromes, thus explaining the differential behavior of SIRT1 in patients with NSTEMI and those with APS. In the production of acute coronary syndromes, vulnerable atherosclerotic plaque prone to ruptures and thrombosis plays a crucial role, and in the production of chronic coronary syndromes, atherosclerosis plays an important role. The decrease in SRTI1 values in patients with APS when compared to the control group suggests SIRT1 protection in atherosclerosis patients.

## 4. Discussion

This manuscript is the first, to our knowledge, to explore the serum dynamics of C5b-9 effectors in acute and chronic coronary syndromes. We report here that serum levels of RGC-32 were significantly lower for the group of patients with unstable angina (the NSTEMI group) than for the control group. Our previous work showed that RGC-32 not only plays a role in *in vitro* endothelial cell proliferation, migration, and cytoskeletal reorganization [31], but it is also expressed by aortic endothelial cells (AECs) and in the media of the atherosclerotic aortic wall, co-localizing with ASMCs, immune-inflammatory cells, and C5b-9 neoantigens [31]. RGC-32 expression increased with the progression of atherosclerosis in both the intima and media of the aortic lesions. Data exist in favor of a pro-atherogenic role for RGC-32: ApoE^−/−^ RGC-32^−/−^ mice display a diminished formation of atherosclerotic lesions when compared to ApoE^−/−^ control mice [19]. RGC-32 participates in the formation of atherosclerotic vascular lesions by mediating aortic SMCs proliferation, migration [20], and differentiation [21].

EC detachment and/or apoptosis as well as VSMC senescence and apoptosis are signature events in the formation of the vulnerable plaques [32]. Our report showing that lower RGC-32 serum values are seen in the NSTEMI group (vulnerable lesions) than in the controls might reflect RGC-32’s influence on both VSMC apoptosis and EC detachment and apoptosis inside such vulnerable plaques. RGC-32 has a binary function in aortic SMC, being involved in sublytic C5b-9-induced cell cycle activation and in regulating TGF-β-induced levels of the extracellular matrix (ECM) components collagen type I, type IV, type V, and fibronectin, as well as SMC differentiation markers, such as myocardin. RGC-32 expression resulted in a phenotype modulation in SMC that can favor ECM deposition and a synthetic phenotype [21]. We have previously substantiated the involvement of RGC-32 in C5b-9-induced angiogenesis: the activation of the Rho-GTPase network, leading to either EC cycle activation via ROCK–Ras–MAPK-Cyclin D1 or focal adhesion of ECs and actin polymerization [31]. Decreased serum levels of RGC-32 in acute coronary syndromes could also be connected to ischemia-driven angiogenesis. *In vitro* work has demonstrated that silencing RGC-32 enhances the proliferation and migration of ECs, thus stimulating angiogenesis [33].

**Figure 7:**
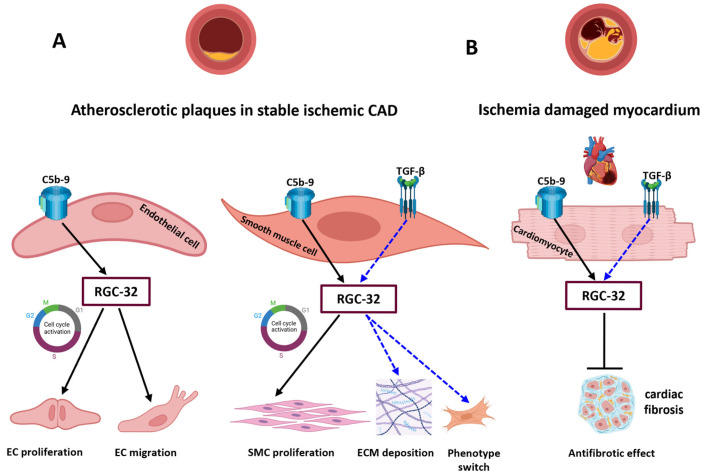
Hypothetical model of the dual role of RGC-32 in the different stages of CAD (adapted from [21]; created in https://BioRender.com). (**A**) In atherosclerotic plaques from stable, chronic, ischemic CAD, RGC-32 plays a pro-atherogenic role; in endothelial cells, RGC-32 mediates complement C5b-9-induced cell cycle activation and migration; similarly, in smooth muscle cells, RGC-32 participates in the C5b-9-induced proliferation of SMCs and TGF-β-dependent phenotype modulation in SMCs, which can favor extracellular matrix ECM deposition and a synthetic phenotype. (**B**) In cardiomyocytes from patients with a prior MI (ischemia-damaged myocardium), the expression of RGC-32, induced by either complement C5b-9 or by TGF-β, could have an antifibrotic, protective effect against cardiac fibrosis.

Among the patients with stable angina (the APS group), our data reveal that higher values of RGC-32 are correlated with higher values of sC5b-9 and a higher number of affected coronary arteries. The notion that RGC-32 serum values are correlated with the number of coronary arteries with stable plaques might relate to RGC-32’s contribution to the formation of the early and intermediate stages of atherosclerotic stable vascular lesions: endothelial dysfunction, fatty streak formation, and fibrous plaque formation (Figure 7). To the same point, our immunohistochemical findings concerning human atherosclerotic aortas confirm that RGC-32 expression increases with the progression of atherosclerosis in both the intima and media of the aortic lesions [31].

We also report that within the APS group, experiencing a previous myocardial infarction was associated with lower RGC-32 values than those of APS patients who had never experienced an AMI. The fibrotic response after an MI is classified into two types: replacement fibrosis (scar formation specifically occurring in the ischemia-injured areas of the myocardium) and reactive fibrosis (occurring in the infarct border zone and the more remote, uninjured myocardium) [34]. TGF-β-induced RGC-32 expression is intimately involved in fibrosis in a variety of tissues [17]. Our finding concerning diminished RGC-32 serum levels in APS patients with a prior MI, and hence a certain degree of myocardial fibrosis, is consistent with the recent experimental data from Luzina and coworkers showing that RGC-32 plays a protective role in pulmonary fibrosis and that a decline in RGC-32 enables collagen accumulation [30]. On a similar note, RGC-32 deficiency in mice has been shown to significantly improve skin and lung fibrosis (sclerosis) by inhibiting the expression of inducible NO synthase (iNOS) and IL-1β in macrophages [35]. Others have demonstrated that RGC-32 expression is upregulated in renal interstitial cells in a mouse model of kidney fibrosis; TGF-β-stimulated RGC-32 expression consistently contributes to *in vitro* fibroblast activation and the epithelial–mesenchymal transition of renal proximal tubular cells [36]. The effects of RGC-32 on fibrosis may be tissue-specific, and, if so, in the case of the myocardium and the lungs, RGC-32 appears to exert an antifibrotic effect (Figure 7).

In the present study, we report that sC5b-9 levels are significantly higher for the NSTEMI group than for the control group and that sC5b-9 levels make it possible to discriminate between acute coronary syndrome patients and controls. Langlois et al. reported that the concentrations of terminal complement complexes (TCCs, which include C5b-9) in plasma increase up to 32-fold by 16 h after an AMI and are only minimally detectable during non-inflammatory myocardial conditions; they found that among the AMI patients, those in the KILLIP III and IV classes of heart failure (indicating a more severe degree of post-MI heart failure [37]) had a peak level of TCCs detected on day 3 post-admission, whereas TCC levels peaked on day 2 for those in KILLIP II and I (less-severe heart failure) [38].

Similarly, several researchers have recently documented higher plasma levels of complement C3, C4, and C5b9 in STEMI patients than in NSTEMI patients, and higher levels in the NSTEMI group than in control subjects [39,40]. Notably, sC5b-9 serum levels in the STEMI and NSTEMI groups peaked at day 3 [39,40]. Others place maximal complement activation even earlier in the time course of an AMI: plasma levels of sC5b9 were found to be significantly higher at admission and at 6 h in the AMI patients than in asymptomatic controls [41]. A comparison between the plasma levels of the proteins and their corresponding levels in the intima and media of the arterial walls indicates a preferential retention of the immune-related proteins in atherosclerotic lesions, suggesting that complement components can also be retained from the plasma [42].

As for the presence of C5b-9 within the ischemic myocardium, when myocardial tissue is damaged during an AMI, it triggers the complement cascade. This activation of complement leads to C5b-9 deposition in the infarcted myocardial tissue, allowing its direct involvement in the damage occurring at the site of infarction [43]. Deposits of the membrane attack complex (MAC)/C5b-9 were observed by immunofluorescence microscopy, and lesions resembling the transmembrane channels of MAC were detected by transmission electron microscopy in areas of tissue injury in myocardial infarction [44].

We also observed that patients in the APS group had significantly lower serum SIRT1 levels than the control group. RGC-32 regulates the expression of SIRT1 in several tissues [17]. Recent experimental studies have demonstrated that SIRT1 activity provides protection against atherosclerosis in ApoE^−/−^ mice, either by modulating macrophage M1 polarization [45] or by reducing hepatic Pcsk9 secretion and enhancing Ldlr expression [46]. SIRT1 mRNA expression levels were found to be significantly lower in AMI patients than in healthy controls [47], and, similarly, diminished monocytic SIRT1 expression was recorded in patients with stable CAD and ACS as compared to subjects without angiographically demonstrable CAD [25]. In contrast, Kilic et al. reported that SIRT1 protein expression levels were increased in patients with coronary artery stenosis as compared to healthy subjects [48]. Within the myocardial tissue, ischemia/reperfusion (I/R) diminished SIRT1 protein and mRNA expression, while the size of myocardial infarction/the ischemic area at risk (AAR) was significantly greater in cardiac-specific Sirt1^−/−^ mice than in control mice [49]. Another very recent *in vitro–in vivo* study on I/R models demonstrated that SIRT1 protects cardiomyocytes from severe oxidative stress via decreased Nrf2 acetylation, thus ameliorating cardiac dysfunction and infarct size during myocardial I/R [50]. Altogether, our observation that subjects with chronic coronary syndromes have lower serum SIRT1 levels than control subjects might be related to the notion that chronic myocardial ischemia reduces SIRT1 levels; furthermore, diminished SIRT1 activity in chronic coronary syndromes leads to diminished protection versus the progression of atherosclerosis. All of this makes SIRT1 an attractive candidate for the pharmacological modulation of sirtuins in the prevention of acute coronary events in patients with high-risk atherosclerotic plaques.

### Study Limitations

One draw-back of this Romanian, observational, population-based study is the relatively small sample size, which limits the statistical power of this study, as calculated in the post hoc analysis. The potential confounding effects of medication should be mentioned. For example, statins, ACE inhibitors, and beta-blockers have known effects on inflammation, endothelial function, and other cardiovascular processes that might interact with C5b-9, RGC-32, and SIRT1. The presence of different comorbidities could have also confounded the results. Although we attempted to account for some of these factors in our statistical analysis, the complexity of these interactions made it difficult to fully disentangle their effects on biomarker levels. We also emphasize that future studies should address these limitations by using larger, multi-center cohorts, controlling for medication use and comorbidities, and employing longitudinal study designs to establish causal relationships.

## 5. Conclusions

sC5b-9 may function as a possible biomarker for myocardial tissue damage in acute coronary syndrome. In such settings, low levels of RGC-32 seem to indicate a protective, antifibrotic function of RGC-32 in the ischemia-damaged myocardium, whereas in stable chronic disease, RGC-32 serum values correlate with the extent of atherosclerotic lesions, advocating for a pro-atherogenic role of RGC-32. Chronic myocardial ischemia scales down SIRT1 protein serum levels, which would support the use of SIRT1-modulating drugs in these patients.

## Figures and Tables

**Table 1:** Main clinical, electrocardiographic, cardiac ultrasound, coronary angiography, blood biochemical parameters and therapeutic data at the start of this study.

Variables	Control Group (n = 21)	APS (n = 41)	NSTEMI (n = 36)	*p*
**Age (years)**	59.8 ± 13.48	68.43 ± 12.4	68.85 ± 12.9	**0.02**
**Men (%)**	28.5	71.79	72.22	**0.002**
**Cardiovascular risk factors:**				
**HTN (%)**	71.42	79.48	91.66	0.49
**Diabetes (%)**	14.28	30.76	30.55	0.27
**Obesity (%)**	57.14	35.89	25	**0.05**
**Smoking (%)**	19.5	15.38	22.22	0.45
**AMI History (%)**	0	58.97	16.66%	0
**Dyslipidemia (%)**	28.57	38.46	47.22	0.30
**Sedentary lifestyle**	38.09	15.79	59.38	**0.008**
**ECG changes**				
**ST segment depression > 1 mm (%) and/or negative T wave**	14.28	46.15	72.22	**<0.001**
**Rhythm or conduction disorders (%)**	9.52	25.64	16.66	0.14
**Echocardiographic parameters:**				
**EF (%)**	54	52.12 ± 4.18	46.46 ± 5.12	0.38
**Ventricular kinetics disorders (n%)**	9.52	51.28	68.33	<**0.001**
**Diastolic dysfunction (n%)**	51	52	60	0.45
**Coronary angiography (n%)**	-	87.8%	88.88%	0.48
**Univascular coronary involvement (%)**	-	55.55%	22.22%	<**0.001**
**Serum biochemical parameters:**				
**Triglycerides (mg/dL)**	124.86 ± 60.18	148.1 ± 82.76	126.57 ± 67.44	0.28
**Cholesterol (mg/dL)**	171.53 ± 10.11	167.9 ± 51	165.57 ± 46.89	0.39
**LDL cholesterol (mg/dL)**	98.12 ± 42.51	106.4 ± 43.44	96 ± 37.09	0.74
**HDL cholesterol (mg/dL)**	53.13 ± 13.67	48 ± 11.36	47.40 ± 22.5	0.03
**Creatinine (mg/dL)**	0.8 ± 0.4	0.86 ± 0.6	0.98 ± 0.1	0.24
**Uric acid (mg/dL)**	5 ± 1.03	5.02 ± 1.84	7.19 ± 5.3	0.25
**Pre-study medication**				
**ACE-I (n%)**	44.33	56.14	51.43	0.23
**Beta-blockers (n %)**	33.33	74.35	47.22	0.007
**Aspirin (n%)**	30	75	100	<0.001
**Statin (n%)**	20	95	70	**<0.001**

Abbreviations used: HTN, hypertension; AMI, acute myocardial infarction; ECG, electrocardiography; EF, ejection fraction of left ventricle; ACE-I, angiotensin-converting enzyme inhibitor. The data represent the mean ± SD or the number of patients expressed as percentages (%).

## Data Availability

The clinical data used to support the findings of this study are included within the article.

## Abbreviations

The following abbreviations are used in this manuscript:

ACE-I	Angiotensin-converting enzyme inhibitor
ACS	Acute coronary syndrome
AMI	Acute myocardial infarction
ANOVA	Analysis of variance
APS	Angina pectoris stable
AUC	Area under the curve
CAD	Coronary artery disease
CO	Control group
Cx	Circumflex artery
DOAJ	Directory of open access journals
ECG	Electrocardiography
ECM	Extracellular matrix
EDD	End-diastolic diameters
EF	Ejection fraction of left ventricle
EKG	Electrocardiogram
ELISAs	Enzyme-linked immunosorbent assays
ESD	End-systolic diameters
GA	Glatiramer acetate
HTN	Hypertension
I/R	Ischemia/reperfusion
IMT	Intima-media thickness
IQR	Interquartile range
LAD	Left Anterior Descending artery
LD	Linear dichroism
LV	Left ventricular
MDPI	Multidisciplinary Digital Publishing Institute
MS	Multiple sclerosis
NO	Nitric oxide
NSTEMI	Non-ST-segment elevation myocardial infarction
PBMCs	Peripheral blood monocytes
RCA	Right Coronary Artery
RGC-32	Response Gene to Complement-32
ROC	Receiver operating characteristic curve
SD	Standard deviation
SIRT1	Sirtuin 1
TLA	Three-letter acronym
UA	Unstable angina
YI	Youden Index
1VD	Single-vessel disease
2VD	Two-vessel disease
3VD	Three-vessel disease
